# Environmental, Family, and Disability Correlates of Flourishing, Anxiety, and Depression Among U.S. Children Aged 6–17 Years: A Cross-Sectional Analysis of the 2023–2024 National Survey of Children’s Health

**DOI:** 10.3390/children13060791

**Published:** 2026-06-06

**Authors:** Joungmin Kim

**Affiliations:** College of Hyangseol Liberal Arts, Healthcare Service School, Soonchunhyang University, Asan 31538, Chungnam, Republic of Korea; nicki123@sch.ac.kr

**Keywords:** child mental health, flourishing, anxiety, depression, ADHD, autism spectrum disorder, bullying, parent mental health, National Survey of Children’s Health, complex sample analysis

## Abstract

**Highlights:**

**What are the main findings?**

- Across nearly 50 million U.S. children aged 6–17 years, parent mental health problems and peer bullying showed the strongest links to all three outcomes—flourishing, anxiety, and depression—surpassing every neighborhood and school factor examined.
- Children with ADHD faced more than fourfold higher odds of anxiety and depression. Although odds-ratio associations of bullying and parental distress were attenuated in ADHD-related analyses, the absolute increase in anxiety and depression with more frequent bullying was larger, not smaller, among children with ADHD.

**What are the implications of the main findings?**

- Routine parent mental health screening at pediatric well-child visits and universal anti-bullying programs are supported as cross-cutting strategies that benefit both typically developing children and those with neurodevelopmental disabilities.
- Children with neurodevelopmental disabilities—particularly ADHD—should receive integrated services combining universal preventive support with disability-specific clinical care, rather than being directed to disability-specific pathways alone.

**Abstract:**

**Background/Objectives:** Children’s mental health and positive development are shaped by family, environmental, and individual factors. Although neurodevelopmental disabilities (NDDs) are well-established correlates of poorer mental health outcomes, few national-scale studies have simultaneously modeled positive (flourishing) and negative (anxiety, depression) outcomes within a unified ecological framework. This study examined how parent mental health, peer victimization, neighborhood and school context, and four NDD diagnoses (autism spectrum disorder [ASD], attention-deficit/hyperactivity disorder [ADHD], developmental delay, and learning disability) are associated with flourishing, current anxiety, and current depression in a national sample of U.S. children aged 6–17 years. **Methods:** Cross-sectional data from the 2023–2024 National Survey of Children’s Health (NSCH; N = 71,172) restricted to ages 6–17 with complete data (unweighted *n* = 64,263; weighted population estimate ≈ 44.6 million children) were analyzed using Complex Sample logistic regression (SPSS 30), accounting for stratified design (state × stratum), household clustering, and sampling weights. Three hierarchical models were estimated for each outcome. NDD-stratified subgroup analyses (*n* = 13,971; weighted ≈ 8.6 million) triangulated moderation findings. Multiple imputation (m = 5) sensitivity analyses confirmed robustness. **Results:** Weighted prevalence was 60.7% for flourishing, 13.2% for current anxiety, and 5.1% for current depression. In Block 2 models, poorer parent mental health and more frequent bullying victimization were robustly associated with all outcomes (flourishing OR = 0.62 and 0.65; anxiety OR = 1.64 and 1.63; depression OR = 1.95 and 1.75; all *p* < 0.001). Supportive neighborhood (flourishing OR = 1.40, depression OR = 0.80) and safe school (flourishing OR = 1.20, anxiety OR = 0.87) were protective. ADHD was the strongest disability-specific correlate (flourishing OR = 0.29; anxiety OR = 4.69; depression OR = 4.27). Three of the twelve interaction terms were significant, all involving ADHD. Relative to children without any NDD, subgroup analyses suggested attenuated associations of parent mental health and bullying with anxiety and depression among children with any NDD (e.g., bullying on anxiety: no-NDD aOR = 1.73 vs. Any-NDD 1.52); however, formal interaction tests identified ADHD as the only significant moderator of these associations. On the absolute-risk scale, however, the increase in internalizing problems with more frequent bullying was larger in children with ADHD. **Conclusions**: Family mental health support and bullying prevention are universally relevant levers for improving children’s mental health and flourishing. Although attenuation of the odds-ratio associations was observed primarily in ADHD-related analyses, specifically for the internalizing outcomes (anxiety and depression), universal anti-bullying and parent mental health interventions remain relevant for children with NDDs, supporting integration into pediatric clinical and public-health programs alongside disability-specific support pathways.

## 1. Introduction

Childhood and adolescence are foundational periods for both psychopathology and positive development. The U.S. Centers for Disease Control and Prevention report that approximately one in five U.S. children aged 3–17 years experiences a current mental, emotional, or behavioral disorder, with anxiety and depression accounting for the largest share of recent increases [1,2]. Concurrently, childhood flourishing operationalized as a composite of curiosity, perseverance, and emotional self-regulation has been promoted by the Maternal and Child Health Bureau as an actionable population indicator, on the premise that positive functioning is not merely the absence of disorder but a distinct construct with its own determinants and implications for policy and practice [3,4].

A growing body of evidence supports an ecological-system view of children’s mental health [5,6], in which family, peer, school, and neighborhood contexts each contribute independently and interactively to risk and resilience. Within the family, parental mental health problems are among the most robust predictors of child anxiety and depression [7,8], with effects mediated by parenting practices, modeling of distress, and direct relational stress. Peer victimization (bullying) similarly shows dose–response associations with internalizing symptoms across cultures [9,10]. At the meso-level, supportive neighborhoods and safe schools have been linked to both a lower prevalence of mental health symptoms and higher flourishing scores [11,12]. However, effect sizes are typically smaller than family-level effects.

Children with neurodevelopmental disabilities (NDDs)—including autism spectrum disorder (ASD), attention-deficit/hyperactivity disorder (ADHD), developmental delay, and learning disabilities—are at substantially elevated risk for co-occurring anxiety and depression [13,14,15], with prevalence estimates two to four times those of typically developing peers. They are also less likely to meet the criteria for flourishing [16]. The preponderance of U.S. evidence indicates that children and youth with disabilities are disproportionately involved in bullying relative to peers without disabilities and that disability-specific school factors such as service intensity, instructional setting, and the restrictiveness of educational placement are correlates of victimization [17,18]. Victimized children with disabilities, in turn, report elevated internalizing symptoms, underscoring an intersection of disability and mental health. A separate question examined here is whether the magnitude of the associations between environmental adversity and child outcomes differs across disability status; this study addresses that question on both the multiplicative (odds ratio) and absolute risk scales.

Three gaps motivate the present analysis. First, prior NSCH-based studies have typically modeled either a positive outcome (flourishing) [19,20] or a negative outcome (anxiety/depression) [21,22], rarely both, leaving open the question of whether identical environmental factors operate symmetrically across the positive–negative spectrum. Second, applications of the NSCH 2023–2024 combined release have been limited; this combined sample provides a substantially larger analytic base than single-year files [23]. Third, although interaction effects of NDD status with environmental factors have been hypothesized, relatively few studies have simultaneously combined design-based moderation testing and stratified subgroup analyses within the same national sample to evaluate convergent evidence. To address these gaps, this study used the 2023–2024 NSCH combined file with proper Complex Sample weighting to (a) estimate weighted prevalence and demographic correlates of flourishing, current anxiety, and current depression in U.S. children aged 6–17 years; (b) examine adjusted associations with parent mental health, peer victimization, sleep adequacy, screen time, food insecurity, and four neighborhood/school context variables, controlling for demographics and four NDD indicators; (c) test whether parent mental health and bullying are differentially associated with outcomes according to ASD or ADHD status; and (d) perform NDD-stratified subgroup analyses to triangulate the interaction findings.

## 2. Materials and Methods

### 2.1. Data Source and Sample

This study analyzed cross-sectional data from the 2023–2024 National Survey of Children’s Health (NSCH), a nationally representative survey of U.S. parents and caregivers conducted by the U.S. Census Bureau on behalf of the Health Resources and Services Administration’s Maternal and Child Health Bureau (HRSA-MCHB) [24,25]. The combined 2023–2024 public-use file contains data on 71,172 children aged 0–17 years; the Child and Adolescent Health Measurement Initiative Data Resource Center (CAHMI DRC)-processed file was used, which provided recoded indicator variables and combined survey weights [26].

The analytic sample was restricted to children aged 6–17 years to align with the universe of the Indicator 2.4 flourishing measure. After excluding cases with missing values in focal predictors (listwise deletion within each model), the effective unweighted sample sizes were 64,263 children for the flourishing model, 63,703 for the anxiety model, and 63,924 for the depression model. Weighted estimates represented approximately 44.6 million U.S. children aged 6–17.

### 2.2. Measures

#### 2.2.1. Outcome Variables

Flourishing was assessed using the CAHMI Indicator 2.4, a parent-report composite of three items capturing curiosity in learning, persistence, and self-regulation [3]. Each item is reported on a four-point scale (Always, Usually, Sometimes, and Never); following the CAHMI construction rule, an item is met when the parent responds in the top categories, and the binary indicator equals 1 only when all three items are met (0 = not flourishing). The binary form was adopted as the primary specification because it is the dominant operationalization in NSCH-based flourishing research and preserves comparability with prior published estimates; a multinomial sensitivity analysis using the traditional three-level coding (at-risk, moderate, and flourishing) is reported in Section 3.6, and it yielded the same direction and ranking of associations. Current anxiety and current depression were derived from the parent-reported “Has a doctor or other health care provider EVER told you that this child has [anxiety/depression]?” items, supplemented by a follow-up regarding current status. Each variable was binary (1 = currently has the condition).

#### 2.2.2. Disability Indicators

The following four NDD indicators were used as predictors: current ASD, ADHD, developmental delay, and learning disability. For subgroup analyses, a composite “Any NDD” variable was created by collapsing across the four NDD indicators; this variable was used solely for stratified subgroup analyses and not for the formal moderation tests, which were conducted separately for ASD and ADHD.

#### 2.2.3. Family and Peer Context

Parent mental health was measured using the CAHMI Indicator 6.2b, a 3-level variable capturing the worst-rated mental health status of co-resident caregivers (1 = excellent or very good; 3 = fair or poor). Bullying victimization was assessed by parent report on a 5-point scale (1 = never to 5 = almost every day in the past 12 months) [9].

#### 2.2.4. Neighborhood and School Context

Three reverse-coded indicators measured perceived neighborhood safety, neighborhood support, and school safety, with higher values indicating safer/more supportive contexts. The counts of neighborhood amenities (0–4: sidewalks, parks, recreation centers, and libraries) and detractors (0–3: litter, poor housing, and vandalism) were used as continuous variables [12].

#### 2.2.5. Health Behaviors

Adequate sleep was operationalized using the American Academy of Pediatrics’ age-appropriate sleep recommendations [27], dichotomized as 1 = adequate vs. 0 = inadequate. Screen time was measured on a 5-level scale (1 = less than 1 h; 5 = 4 or more hours per weekday) [28]. Food insecurity was a 4-level scale (1 = always afford to 4 = often could not afford enough food) [29].

#### 2.2.6. Demographic Covariates

Demographic variables included child age in years (continuous, 6–17), child sex (1 = female, 0 = male), race/ethnicity (dummy-coded as Hispanic, non-Hispanic Black, and non-Hispanic multi-racial, with non-Hispanic White as reference), and family poverty level (1 = 0–99% of federal poverty line to 4 = 400% or above).

### 2.3. Statistical Analysis

All analyses were conducted in IBM SPSS Statistics 30 using the Complex Sample module (CSPLAN, CSDESCRIPTIVES, CSTABULATE, CSGLM, and CSLOGISTIC procedures) to produce design-consistent point estimates and standard errors [30]. The Complex Sample plan specified two stratification variables (state of residence and sampling stratum), which yielded 102 strata; household identifier as the cluster variable; the 2023–2024 combined child weight; and the with-replacement variance estimator recommended by HRSA for the public-use NSCH file.

Three hierarchical logistic regression models were estimated for each outcome. Block 1 included demographic covariates only. Block 2 added all environmental, family, peer, and disability variables. Block 3 added the following four interaction terms: parent mental health × ASD, parent mental health × ADHD, bullying × ASD, and bullying × ADHD; both parent mental health and bullying were mean-centered before forming product terms [31].

To complement the Block 3 interaction tests and provide convergent evidence of potential effect modification, post hoc subgroup analyses were performed by re-estimating the Block 2 model separately within the Any-NDD subgroup. Because the SPSS multiple imputation procedure does not currently integrate with the Complex Sample logistic regression module, a sensitivity analysis was additionally conducted using R via the mice [32] and survey [33] packages, with imputation-pooled estimates obtained via mitools (Rubin’s rules) [34]; results did not differ substantively from complete-case estimates (all OR differences < 5%).

Pseudo-R^2^ values (Cox & Snell, Nagelkerke, McFadden) were reported as descriptive indicators of model fit and explanatory variance, and Wald F-tests of model effects were used to evaluate predictor significance. All *p*-values are two-sided, with significance set at *p* < 0.05.

### 2.4. Ethical Considerations

Analyses used de-identified public-use NSCH data; no institutional review board approval was required.

## 3. Results

### 3.1. Sample Characteristics

The weighted analytic sample represented approximately 44.6 million U.S. children aged 6–17 years. Mean age was 12.06 years (SD = 3.49); 51.3% were male and 48.7% female. Race/ethnicity distribution was 51.6% non-Hispanic White, 27.0% Hispanic, 12.4% non-Hispanic Black, and 12.6% non-Hispanic multi-racial or other. Of the 71,172 children in the full file, 21.1% (*n* = 15,044) had at least one NDD; after model-specific listwise deletion of cases with missing predictor data, 13,971 children were included in the NDD subgroup analyses.

Weighted prevalence estimates indicated that flourishing was substantially more common than current anxiety or depression, occurring in 60.7% (95% CI: 60.0–61.4) of children, whereas current anxiety and depression affected 13.2% (95% CI: 12.8–13.7) and 5.1% (95% CI: 4.8–5.4), respectively. The weighted prevalence of NDDs in the analytic sample was: ADHD 13.3%, ASD 4.5%, learning disability 9.1%, and developmental delay 5.8% (Table 1).

### 3.2. Hierarchical Logistic Regression Results: Flourishing

**Figure 1:**
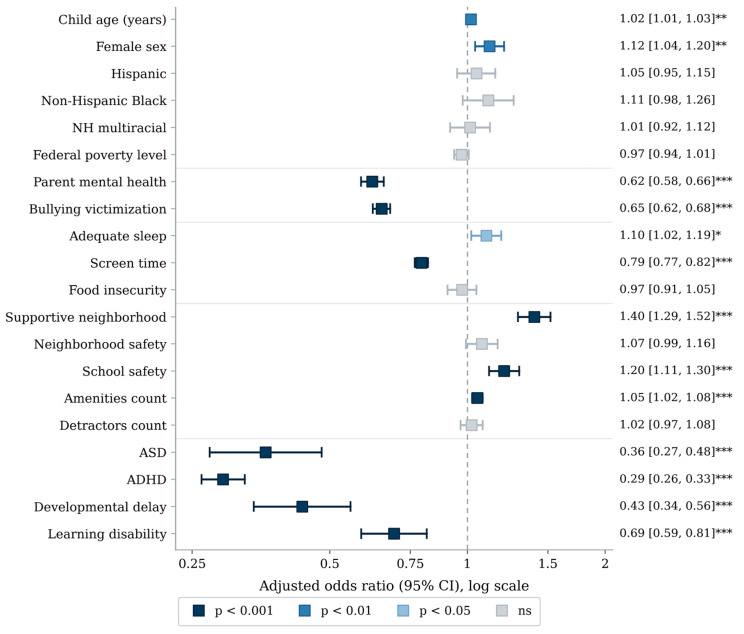
Adjusted odds ratios (aORs) and 95% confidence intervals for predictors of flourishing among U.S. children aged 6–17 years (NSCH 2023–2024). Estimates from Block 2 Complex Sample logistic regression (unweighted n = 64,263; weighted population estimate ≈ 44.6 million). Color reflects the statistical significance level. Vertical dashed reference line: aOR = 1.0. * *p* < 0.05, ** *p* < 0.01, *** *p* < 0.001.

After adjustment for demographic, behavioral, neighborhood, school, and disability variables, poorer parent mental health (aOR = 0.62, 95% CI [0.59, 0.66]) and more frequent bullying victimization (aOR = 0.65, 95% CI [0.62, 0.68]) were strongly associated with lower odds of flourishing. Among the disability indicators, ADHD showed the strongest negative association with flourishing (aOR = 0.29, 95% CI [0.26, 0.33]), followed by ASD (aOR = 0.36, 95% CI [0.27, 0.48]). Adequate sleep, supportive neighborhoods, school safety, and neighborhood amenities were positively associated with flourishing (Table 2; Figure 1).

### 3.3. Hierarchical Logistic Regression Results: Current Anxiety

**Figure 2:**
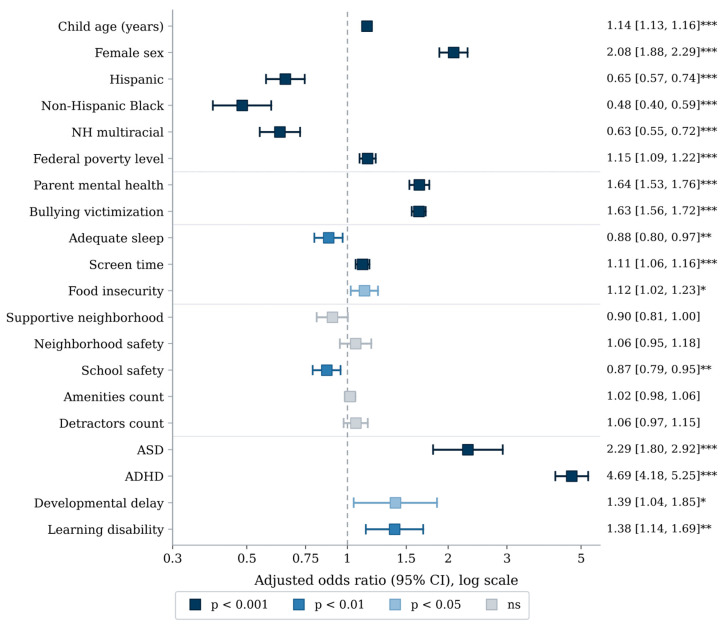
Adjusted odds ratios for current anxiety. NSCH 2023–2024 (n = 63,703). Vertical dashed reference line: aOR = 1.0. * *p* < 0.05, ** *p* < 0.01, *** *p* < 0.001.

For current anxiety, poorer parent mental health (aOR = 1.64, 95% CI [1.53, 1.76]) and more frequent bullying victimization (aOR = 1.63, 95% CI [1.56, 1.72]) remained among the strongest correlates. ADHD was associated with substantially elevated odds of anxiety (aOR = 4.69, 95% CI [4.18, 5.25]), whereas ASD was associated with approximately twofold higher odds (aOR = 2.29, 95% CI [1.80, 2.92]). Adequate sleep and school safety were associated with lower odds of anxiety (Table 3; Figure 2).

### 3.4. Hierarchical Logistic Regression Results: Current Depression

**Figure 3:**
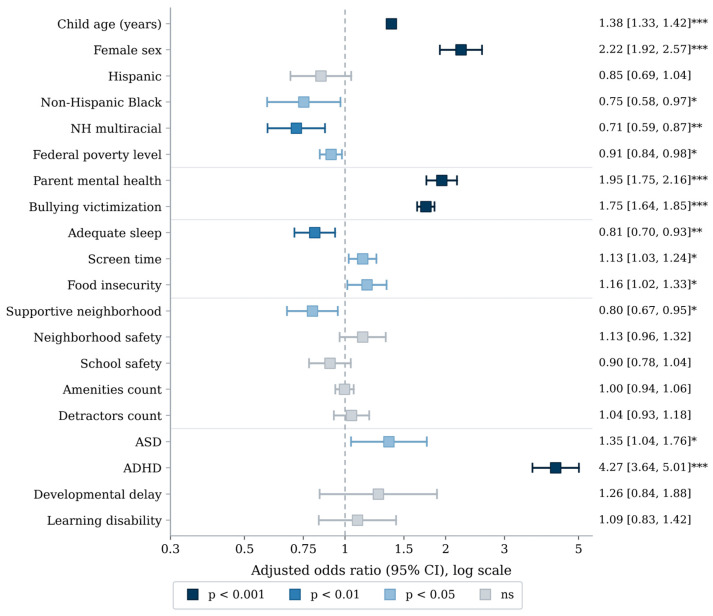
Adjusted odds ratios for current depression. NSCH 2023–2024 (n = 63,924). Vertical dashed reference line: aOR = 1.0. * *p* < 0.05, ** *p* < 0.01, *** *p* < 0.001.

Current depression showed a pattern broadly similar to that observed for anxiety. Poorer parent mental health (aOR = 1.95, 95% CI [1.75, 2.16]) and more frequent bullying victimization (aOR = 1.75, 95% CI [1.64, 1.85]) were strongly associated with higher odds of depression. ADHD remained the strongest disability-specific correlate (aOR = 4.27, 95% CI [3.64, 5.01]), whereas developmental delay and learning disability were not independently associated with depression after adjustment for the full set of covariates (Table 4; Figure 3).

### 3.5. Diagnosis-Specific Models

To examine the heterogeneity of the NDD group, the full Block 2 model was re-estimated separately for children with ADHD only (n = 10,087–10,269; population ≈ 5.9–6.0 million) and for children with ASD only (n = 3052–3113; population ≈ 1.9–2.0 million), excluding overlapping diagnoses. Within the ADHD subgroup, parent mental health and bullying remained significant correlates of anxiety (aOR = 1.43 [1.26, 1.61] and 1.51 [1.40, 1.63], respectively) and depression (aOR = 1.84 [1.57, 2.16] and 1.58 [1.46, 1.71]). Female sex was a particularly strong correlate of internalizing symptoms in the ADHD subgroup (anxiety aOR = 1.99 [1.68, 2.35]; depression aOR = 2.02 [1.65, 2.47]), and perceived school safety remained protective (anxiety aOR = 0.79 [0.68, 0.91]; depression aOR = 0.84 [0.71, 0.99]). Within the ASD subgroup, parent mental health and bullying were similarly associated with anxiety and depression, though with wider confidence intervals (Table 5). All results are reported in Table 5 alongside the no-NDD, Any-NDD, and full-sample estimates.

Of the 12 formal interaction tests, three were statistically significant, all involving ADHD. Specifically, the associations of parent mental health problems and bullying victimization with anxiety and depression were weaker on the multiplicative (odds-ratio) scale among children with ADHD than among children without ADHD—specifically, parent MH × ADHD on anxiety (aOR = 0.856, 95% CI: 0.746–0.983, *p* = 0.028); bullying × ADHD on anxiety (aOR = 0.878, 95% CI: 0.795–0.970, *p* = 0.010); and bullying × ADHD on depression (aOR = 0.831, 95% CI: 0.738–0.935, *p* = 0.002). All other interactions involving parent MH × ASD, bullying × ASD, and the various interactions on the flourishing outcome were nonsignificant (all *p* > 0.15).

### 3.6. Subgroup Analyses by Neurodevelopmental Disability Status

To triangulate the Block 3 interaction findings, the Block 2 model was re-estimated separately within children with at least one NDD (*n* = 13,971; weighted population ≈ 8.6 million). All three NDD-subgroup models showed adequate fit (Cox & Snell R^2^: flourishing 0.116, Nagelkerke 0.168; anxiety 0.128, Nagelkerke 0.174; and depression 0.148, Nagelkerke 0.259). All key environmental predictors retained statistical significance with directionally consistent effects (Table 5).

Relative to children without any NDD (the appropriate reference group rather than the full sample, which itself includes children with NDDs), the parent mental health and bullying associations with the internalizing outcomes were consistently smaller in the Any-NDD subgroup. For anxiety, the parent mental health association was attenuated (no-NDD aOR = 1.78 vs. Any-NDD aOR = 1.45), as was the bullying association (1.73 vs. 1.52); for depression, both were attenuated (parent mental health aOR = 2.04 vs. 1.81; bullying aOR = 1.96 vs. 1.59). For flourishing, by contrast, the bullying association was not attenuated (no-NDD 0.65 vs. Any-NDD 0.62), so the attenuation is restricted to the internalizing outcomes. These patterns were broadly consistent with the Block 3 interaction results; however, formal moderation was statistically significant only for ADHD, whereas the subgroup analyses suggested a more general pattern of attenuation among children with NDDs (Table 5).

Block 2 models were also estimated within the no-NDD subgroup (analytic n = 51,313–51,707) to provide the reference contrast, and within the ADHD-only (n = 10,087–10,269) and ASD-only (n = 3052–3113) subgroups to address diagnostic heterogeneity; all models converged and are reported in Table 5. Survey-weighted absolute probabilities of anxiety and depression across bullying levels by ADHD status are reported in Table 6, and a severity- and treatment-adjusted sensitivity analysis is reported in Section 3.7.

### 3.7. Severity- and Treatment-Adjusted Sensitivity Analysis

To address the concern that diagnoses are treated as binary, ignoring symptom severity, comorbidities, and treatment, a severity- and treatment-adjusted sensitivity analysis was conducted within the Any-NDD subgroup (*n* = 13,733–13,870; population ≈ 8.4–8.6 million). Parental ratings of current ADHD severity (1 = mild to 3 = severe), ASD severity, and binary indicators of ADHD/ASD medication use and behavioral-treatment receipt were added as covariates to the Block 2 models for anxiety and depression. After adjustment for severity and treatment status, parent mental health (anxiety aOR = 1.41 [1.26, 1.58]; depression aOR = 1.74 [1.51, 2.02]) and bullying (anxiety aOR = 1.41 [1.32, 1.51]; depression aOR = 1.49 [1.39, 1.60]) remained statistically significant. Severity itself was independently associated with outcomes (e.g., ADHD severity on depression: aOR = 1.38 [1.17, 1.62]), confirming its status as a meaningful covariate, yet its inclusion did not substantially attenuate the focal associations. These results confirm the robustness of the parent mental health and bullying associations within the NDD subgroup beyond simple diagnostic status.

## 4. Discussion

This study makes three specific incremental advances to the NSCH and child mental health literature. First, it is the first analysis of the combined NSCH 2023–2024 file to examine flourishing, anxiety, and depression simultaneously within a unified ecological framework, allowing direct evaluation of whether family, peer, school, and neighborhood factors operate symmetrically across positive and negative outcomes. Second, it employs multiple convergent methods—formal interaction tests, stratified subgroup models, diagnosis-specific models (ADHD-only, ASD-only), and absolute-risk comparisons—to triangulate effect modification, rather than relying on interaction tests alone; this triangulation yields more stable and policy-relevant inferences. Third, the absolute-risk analysis (Table 6) demonstrates that odds-ratio attenuation on the multiplicative scale does not reflect smaller behavioral burden in children with ADHD, but rather odds-ratio compression at higher baseline prevalence—a finding with direct implications for clinical interpretation and public-health policy. While the study corroborates well-established correlates, these three contributions address a gap in how environmental effects are characterized across disability subgroups and in how odds ratios should be interpreted in populations with high baseline prevalence.

### 4.1. Principal Findings

Using the 2023–2024 NSCH combined file with Complex Sample weighting (≈44.6 million children represented), this study identified five key patterns. First, parent mental health problems and peer bullying victimization were consistently associated with all three outcomes, flourishing, anxiety, and depression, with effect sizes (in absolute terms) exceeding those of any neighborhood or school context variable. Second, supportive neighborhood and school safety were associated with higher odds of flourishing and lower odds of depression. In contrast, their associations with anxiety were not statistically significant after adjustment for family and peer factors. Third, ADHD showed the largest magnitude of association among neurodevelopmental conditions, particularly for anxiety (aOR = 4.69) and depression (aOR = 4.27), and was followed by ASD (anxiety aOR = 2.29; depression aOR = 1.35); both NDDs were also clearly associated with reduced flourishing. Fourth, three of twelve formal moderation tests reached significance, all involving ADHD and all in the same direction: parent mental health × ADHD on anxiety (aOR = 0.86, *p* = 0.028), bullying × ADHD on anxiety (aOR = 0.88, *p* = 0.010), and bullying × ADHD on depression (aOR = 0.83, *p* = 0.002), indicating that the multiplicative associations of family- and peer-level adversity with anxiety and depression were attenuated among children with ADHD. Fifth, subgroup analyses using the no-NDD reference group indicated that environmental correlates remained statistically significant within the NDD subgroup, although the odds ratios for the internalizing outcomes were smaller (e.g., parent mental health on anxiety: no-NDD aOR = 1.78 vs. Any-NDD 1.45; bullying on anxiety: 1.73 vs. 1.52; and bullying on depression: 1.96 vs. 1.59), whereas the flourishing–bullying association was not attenuated (0.65 vs. 0.62); the formal moderation tests were significant only for ADHD.

### 4.2. Family Mental Health and Bullying as Central Correlates

The consistent associations of parent mental health and bullying victimization across all outcomes are aligned with prior research emphasizing the role of proximal interpersonal environments in child mental health [5,6]. Each one-step increase on the three-level parent mental health scale was associated with a 38% reduction in odds of flourishing (aOR = 0.62), a 64% increase in odds of anxiety (aOR = 1.64), and a 95% increase in odds of depression (aOR = 1.95). Parallel effects for bullying victimization (per one-step increase on the five-level scale) were a 35% reduction in flourishing odds (aOR = 0.65), a 63% increase in anxiety odds (aOR = 1.63), and a 75% increase in depression odds (aOR = 1.75; all *p* < 0.001). These magnitudes substantially exceed those observed for neighborhood/school context variables, school behavioral indicators, and even most demographic covariates, reinforcing that family and peer interpersonal contexts represent the most central modifiable correlates of both positive and negative child mental health outcomes identified in this analysis [7,9,10].

### 4.3. Interpretation of Interaction and Subgroup Findings

The Block 3 interaction findings (nine of 12 nonsignificant, three of 12 significant, and all involving ADHD) and the post hoc NDD subgroup analyses jointly inform the question of whether environmental factors operate similarly across disability status.

On the surface, the largely null Block 3 interactions might suggest that environmental associations are broadly similar across NDD and non-NDD groups. However, the subgroup analyses, contrasting children with NDDs against the appropriate no-NDD reference group, reveal a more nuanced picture as follows: parent mental health and bullying victimization remained statistically significant correlates of all three outcomes within the NDD subgroup, but with smaller odds ratios for the internalizing outcomes relative to no-NDD children (anxiety: parent mental health 1.78 → 1.45, bullying 1.73 → 1.52; depression: parent mental health 2.04 → 1.81, bullying 1.96 → 1.59). For flourishing, the bullying association was not attenuated (0.65 vs. 0.62), so the attenuation is specific to anxiety and depression. Importantly, the formal interaction terms were significant only for ADHD (three of 12), so the moderation is restricted to ADHD and is not generalized to ASD, developmental delay, or learning disability. Critically, the smaller odds ratios do not indicate a smaller behavioral burden: the absolute-risk estimates (Table 6) show that the absolute-risk increases are larger in children with ADHD (anxiety +41.5 vs. +28.9 percentage points; depression +32.8 vs. +19.8 pp), indicating odds-ratio compression rather than reduced environmental sensitivity. The attenuation on the multiplicative scale is therefore best read as one of several non-exclusive phenomena—odds-ratio compression, shared-source variance, and a possible greater relative contribution of neurobiological pathways in ADHD—none of which can be adjudicated by the present cross-sectional design.

Taken together, these findings suggest attenuation of the multiplicative associations primarily for ADHD and internalizing outcomes; however, formal moderation analyses were statistically significant only in ADHD-related analyses. Three non-exclusive explanations are plausible. First, the odds ratio is compressed at the higher baseline prevalences seen in ADHD, so the same—or a larger—absolute-risk gradient yields a smaller odds ratio (Table 6). Second, because all measures derive from a single parent informant, shared-source variance may compress within-subgroup odds ratios. Third, neurobiological and clinical pathways may contribute relatively more to ADHD, reducing the marginal multiplicative contribution of psychosocial exposures. These explanations are not mutually exclusive and cannot be adjudicated in cross-sectional NSCH data; the policy-relevant point, robust across all three, is that the key environmental correlates remain statistically and clinically significant within NDD subgroups, so universal anti-bullying and parent mental health programs remain relevant for children with NDDs.

### 4.4. Strengths

Strengths of this study include (a) the use of a large, nationally representative dataset (combined 2023–2024 NSCH; weighted population estimate ≈ 44.6 million U.S. children); (b) application of Complex Sample weighting, producing design-consistent point estimates and standard errors; (c) parallel modeling of multiple outcomes, allowing direct comparison of effect directions and magnitudes across the positive–negative spectrum; (d) inclusion of all four NSCH-measured neurodevelopmental conditions within a single analytic framework rather than focusing on a single disability; (e) formal moderation tests with mean-centered predictors to reduce non-essential collinearity in interaction terms; and (f) a dual-strategy approach combining formal interaction tests with stratified subgroup analyses, providing convergent evidence about disability-related effect modification beyond what either method could establish alone.

### 4.5. Limitations

Single-informant source bias is a critical limitation. Because all predictor and outcome measures derive from parent report, shared-source variance may inflate associations, particularly those between parent mental health and child psychological outcomes. A parent experiencing psychological distress may report both their own mental health problems and their child’s anxiety/depression more negatively than an independent observer would, inducing spurious covariation. This concern is most acute for the parent mental health associations (which show the largest odds ratios) and is substantially reduced for the more behaviorally observable or externally verified variables (bullying victimization reported by parents but grounded in peer interactions; neighborhood detractors representing concrete environmental features; and school safety reflecting institutional policies). Multi-informant designs incorporating teacher or child self-reports, or administrative data on school absences and disciplinary incidents, would strengthen causal inference in future research. Accordingly, replication with independent ascertainment of outcomes is encouraged before clinical translation of the parent mental health findings.

#### 4.5.1. Cross-Sectional Design Precludes Causal Inference

Bidirectional pathways are particularly plausible for parent mental health and child outcomes [7], and similarly for bullying victimization, given that emotionally distressed children may be more frequently targeted as well as harmed by victimization. Future longitudinal analyses using the panel components of the NSCH or external datasets such as the Adolescent Brain Cognitive Development Study would clarify temporal precedence and adjudicate among competing causal models.

#### 4.5.2. Reliance on Parent-Proxy Reporting and Diagnostic Ascertainment

Shared-source variance may inflate associations between parent-reported parent mental health and parent-reported child outcomes. The doctor-told format of the anxiety and depression measures captures clinical diagnostic ascertainment rather than symptom prevalence, which may bias estimates downward in populations with reduced healthcare access [2]. The protective race/ethnicity associations observed for anxiety and depression most plausibly reflect a combination of true prevalence differences, lower diagnostic ascertainment among historically underserved populations due to healthcare access barriers, cultural variation in symptom recognition, and stigma-related underreporting, rather than genuinely lower psychopathology among minoritized children. Future work should triangulate parent-reported outcomes with structured diagnostic assessments or adolescent self-reports when feasible.

#### 4.5.3. Missing Data and Variance Estimation

Approximately 7% of cases were excluded due to listwise deletion. Sensitivity analysis using multiple imputation (*m* = 5, fully conditional specification with predictive mean matching) implemented in R version 4.6.0 via the *mice* package [32] integrated with Complex Sample logistic regression via the *survey* [33] and *mitools* [34] packages yielded estimates that did not differ substantively from complete-case results (all OR differences < 5%; no change in significance for any predictor with original *p* < 0.01). The SPSS multiple imputation procedure does not currently integrate directly with the Complex Sample logistic regression module, motivating the R-based sensitivity analysis. The with-replacement Complex Sample variance estimator employed is the HRSA-recommended approach for the public-use NSCH file [25].

#### 4.5.4. Limited Depression Outcome Sample and Convergence Issues in No-NDD Subgroup

Current depression is rare in this age range (weighted prevalence 5.1%), which yields wider confidence intervals for depression-model estimates than for the more common flourishing and anxiety outcomes. In the planned no-NDD subgroup analyses, the depression model exhibited convergence difficulties owing to very low outcome prevalence (<4%), and potentially unreliable estimates were not reported. The NDD subgroup, by contrast, supported stable convergence across all three outcomes, thereby allowing the within-NDD effect-modification analyses summarized in Section 3.6.

#### 4.5.5. Linear and Additive Modeling Assumptions

The hierarchical logistic regression assumed linearity in the logit for ordinal and continuous predictors. Sensitivity analyses using categorical recoding did not reveal meaningful nonlinear patterns, but more sophisticated approaches, such as restricted cubic splines, could be explored in future work.

#### 4.5.6. Generalizability and Sampling Frame

The NSCH samples noninstitutionalized U.S. children with valid mailing addresses; consequently, children in foster care, group homes, residential treatment, and juvenile justice settings—who experience disproportionately high mental health burden—are underrepresented [25]. Estimates, therefore, likely underestimate the true population mental health burden among the most vulnerable U.S. children.

#### 4.5.7. Modest Magnitude of Significant Interactions

The three significant interactions (aORs 0.83–0.88) were modest in magnitude despite reaching conventional statistical significance. Although consistent with attenuation of the multiplicative associations among children with ADHD and corroborated by subgroup analyses, these interactions require replication in independent samples and longitudinal designs before clinical translation; as shown by the absolute-risk estimates, they do not indicate a reduced behavioral burden. The relatively small effect sizes also limit the practical utility of these interactions for individual-level risk stratification.

### 4.6. Future Directions

Future research should: (a) replicate these findings using longitudinal designs to establish temporal precedence; (b) examine whether the bullying-by-ADHD interaction generalizes to other markers of psychosocial adversity such as adverse childhood experiences [4]; (c) test mediation pathways linking parent mental health to child outcomes via parenting practices; (d) examine whether pediatric clinical interventions targeting parent mental health and structured bullying-prevention programs in schools yield differential benefits across NDD subgroups; and (e) extend the present subgroup framework to disability-specific subpopulations (e.g., ASD-only, ADHD-only) sufficiently powered to detect subtle moderation patterns that the broader NDD aggregate may obscure.

### 4.7. Policy and Clinical Implications

The present findings have several implications for pediatric mental health practice and public-health policy.

First, the absolute burden associated with bullying was greater among children with ADHD, despite the smaller odds-ratio associations observed in the moderation models. This distinction is important because odds ratios can appear attenuated when baseline prevalence is already elevated. The findings suggest that environmental adversities such as bullying remain highly relevant for children with ADHD and that improvements in these environments may have the potential to produce substantial absolute reductions in internalizing problems.

Second, universal interventions remain important for children both with and without neurodevelopmental disabilities. Parent mental health problems and bullying victimization were consistently associated with flourishing, anxiety, and depression across analytic models, including disability-specific subgroup analyses. These findings support the continued integration of parent mental health screening [35] and anti-bullying initiatives within pediatric, school, and community-based services rather than limiting intervention efforts to disability-specific programs alone.

Third, ADHD was the only neurodevelopmental condition that demonstrated statistically significant moderation effects. No significant moderation effects were observed for ASD, developmental delay, or learning disability. Although the mechanisms underlying these findings cannot be determined from cross-sectional data, the results suggest that children with ADHD may differ from other neurodevelopmental groups in the way environmental factors relate to internalizing outcomes. Enhanced monitoring of bullying experiences, family mental health context, and emotional well-being may therefore be particularly warranted for this population within universal prevention frameworks.

Taken together, these findings support a dual approach that combines population-level prevention strategies with targeted support for children at elevated developmental risk.

## 5. Conclusions

In a cross-sectional, nationally representative sample of approximately 44.6 million U.S. children aged 6–17 years, parent mental health problems and peer bullying victimization were the most consistent and clinically substantial environmental correlates of children’s flourishing, anxiety, and depression, operating in expected directions for both positive and negative outcomes. ADHD was the strongest disability-specific correlate. Both formal interaction tests and stratified subgroup analyses suggested that, on the odds-ratio scale, the associations of environmental adversity with the internalizing outcomes were attenuated among children with ADHD specifically; on the absolute-risk scale; however, the burden associated with frequent bullying was larger, not smaller, in this subgroup, suggesting that the attenuation may reflect a scale phenomenon rather than evidence that environmental adversity is less relevant for children with ADHD.

Within children with NDDs, parent mental health, bullying victimization, supportive neighborhood, and school safety all retained statistical and clinical significance, supporting the applicability of family- and school-focused interventions even within populations at elevated developmental risk. These findings support consideration of routine parent mental health screening during pediatric well-child visits, universal anti-bullying programs, and enhanced monitoring of mental health comorbidities among children with ADHD.

Given the cross-sectional design, longitudinal replication is needed to establish temporal precedence and to determine whether interventions targeting these correlates are associated with reductions in psychopathology and improvements in flourishing across disability subgroups.

## Figures and Tables

**Table 1:** Weighted sample characteristics (NSCH 2023–2024, ages 6–17 years).

Characteristic	Weighted % or M (SE)	95% CI	Unweighted N
***Outcomes***			
Flourishing (all 3 items)	60.7%	60.0–61.4	70,527
Current anxiety	13.2%	12.8–13.7	70,406
Current depression	5.1%	4.8–5.4	70,715
***Disability***			
Any NDD	21.1%	20.5–21.7	15,044
ADHD (current)	13.3%	12.9–13.7	70,449
Autism spectrum disorder	4.5%	4.3–4.7	70,764
Developmental delay	5.8%	5.5–6.1	70,766
Learning disability	9.1%	8.8–9.4	70,768
***Demographics***			
Age (years), M (SD)	12.06 (3.49)	—	71,172
Female sex	48.7%	48.0–49.4	71,172
Hispanic	27.0%	26.4–27.6	71,172
Non-Hispanic Black	12.4%	11.9–12.9	71,172
Non-Hispanic multi-racial/other	12.6%	12.2–13.1	71,172
***Family/context***			
Parent mental health (1–3, higher = worse), M (SD)	1.49 (0.67)	—	69,075
Bullying victimization (1–5), M (SD)	1.66 (0.93)	—	70,115
Adequate sleep	68.1%	67.4–68.8	70,259
Supportive neighborhood (yes)	61.3%	60.5–62.1	68,714
Safe school (high)	66.6%	65.9–67.3	68,894

Note. Weighted estimates use the NSCH 2023–2024 combined child weight (fwc_2324) with stratification (state × sampling stratum) and household clustering. Percentages and means are weighted; sample sizes are unweighted. SE = standard error; CI = confidence interval; M = mean; SD = standard deviation. NDD = neurodevelopmental disability.

**Table 2:** Flourishing Block 2 adjusted odds ratios (n = 64,263).

Predictor	B	SE	aOR	95% CI	*p*
***Demographics***					
Child age (years)	0.017	0.006	1.017	[1.006, 1.029]	0.002
Female sex	0.111	0.037	1.118	[1.040, 1.202]	0.003
Hispanic	0.045	0.049	1.046	[0.950, 1.151]	0.359
Non-Hispanic Black	0.105	0.065	1.110	[0.977, 1.262]	0.108
Non-Hispanic multi-racial	0.013	0.051	1.013	[0.916, 1.120]	0.803
Federal poverty level	−0.030	0.019	0.970	[0.935, 1.007]	0.114
***Family/peer***					
Parent mental health	−0.479	0.029	0.619	[0.585, 0.656]	<0.001
Bullying victimization	−0.432	0.022	0.649	[0.621, 0.678]	<0.001
***Health behaviors***					
Adequate sleep	0.095	0.039	1.100	[1.020, 1.186]	0.014
Screen time	−0.232	0.017	0.793	[0.767, 0.820]	<0.001
Food insecurity	−0.028	0.037	0.973	[0.905, 1.046]	0.454
***Neighborhood/school***					
Supportive neighborhood	0.336	0.042	1.400	[1.290, 1.520]	<0.001
Neighborhood safety	0.072	0.041	1.075	[0.993, 1.164]	0.075
School safety	0.185	0.039	1.203	[1.115, 1.298]	<0.001
Amenities count	0.050	0.014	1.051	[1.024, 1.080]	<0.001
Detractors count	0.021	0.029	1.021	[0.966, 1.080]	0.463
***Disability***					
ASD (current)	−1.016	0.143	0.362	[0.273, 0.480]	<0.001
ADHD (current)	−1.229	0.056	0.292	[0.262, 0.326]	<0.001
Developmental delay	−0.832	0.124	0.435	[0.341, 0.555]	<0.001
Learning disability	−0.370	0.084	0.691	[0.586, 0.815]	<0.001

Note. Reference category for outcome = not flourishing. Reference category for sex = male; reference category for race/ethnicity = non-Hispanic White. aOR = adjusted odds ratio from Complex Sample logistic regression.

**Table 3:** Current anxiety Block 2 adjusted odds ratios (n = 63,703).

Predictor	B	SE	aOR	95% CI	*p*
***Demographics***					
Child age (years)	0.135	0.008	1.144	[1.126, 1.163]	<0.001
Female sex	0.731	0.050	2.076	[1.884, 2.288]	<0.001
Hispanic	−0.427	0.068	0.652	[0.571, 0.745]	<0.001
Non-Hispanic Black	−0.726	0.103	0.484	[0.396, 0.592]	<0.001
Non-Hispanic multi-racial	−0.465	0.071	0.628	[0.547, 0.722]	<0.001
Federal poverty level	0.139	0.028	1.149	[1.088, 1.215]	<0.001
***Family/peer***					
Parent mental health	0.496	0.035	1.641	[1.533, 1.758]	<0.001
Bullying victimization	0.491	0.025	1.634	[1.557, 1.716]	<0.001
***Health behaviors***					
Adequate sleep	−0.131	0.050	0.878	[0.796, 0.967]	0.009
Screen time	0.104	0.024	1.110	[1.058, 1.164]	<0.001
Food insecurity	0.116	0.048	1.124	[1.023, 1.234]	0.015
***Neighborhood/school***					
Supportive neighborhood	−0.105	0.055	0.901	[0.809, 1.003]	0.056
Neighborhood safety	0.056	0.055	1.058	[0.949, 1.179]	0.310
School safety	−0.143	0.049	0.867	[0.787, 0.954]	0.003
Amenities count	0.018	0.019	1.018	[0.982, 1.057]	0.332
Detractors count	0.057	0.042	1.059	[0.974, 1.151]	0.177
***Disability***					
ASD (current)	0.830	0.123	2.292	[1.803, 2.915]	<0.001
ADHD (current)	1.545	0.058	4.687	[4.183, 5.251]	<0.001
Developmental delay	0.330	0.146	1.392	[1.044, 1.854]	0.024
Learning disability	0.324	0.101	1.383	[1.135, 1.686]	0.001

Note. Reference category for outcome = no current anxiety. n = 63,703 unweighted.

**Table 4:** Current depression Block 2 adjusted odds ratios (n = 63,924).

Predictor	B	SE	aOR	95% CI	*p*
***Demographics***					
Child age (years)	0.319	0.016	1.375	[1.333, 1.418]	<0.001
Female sex	0.799	0.074	2.223	[1.923, 2.570]	<0.001
Hispanic	−0.168	0.107	0.846	[0.686, 1.043]	0.117
Non-Hispanic Black	−0.285	0.129	0.752	[0.584, 0.969]	0.027
Non-Hispanic multi-racial	−0.336	0.101	0.714	[0.586, 0.871]	0.001
Federal poverty level	−0.098	0.039	0.907	[0.840, 0.979]	0.012
***Family/peer***					
Parent mental health	0.666	0.054	1.947	[1.752, 2.163]	<0.001
Bullying victimization	0.557	0.031	1.745	[1.644, 1.853]	<0.001
***Health behaviors***					
Adequate sleep	−0.209	0.072	0.811	[0.705, 0.934]	0.004
Screen time	0.121	0.048	1.128	[1.026, 1.241]	0.013
Food insecurity	0.151	0.069	1.163	[1.016, 1.331]	0.029
***Neighborhood/school***					
Supportive neighborhood	−0.225	0.089	0.798	[0.670, 0.951]	0.012
Neighborhood safety	0.121	0.081	1.129	[0.963, 1.323]	0.134
School safety	−0.104	0.074	0.901	[0.780, 1.041]	0.159
Amenities count	−0.004	0.032	0.996	[0.935, 1.061]	0.907
Detractors count	0.044	0.062	1.045	[0.926, 1.181]	0.476
***Disability***					
ASD (current)	0.302	0.133	1.353	[1.042, 1.756]	0.023
ADHD (current)	1.452	0.082	4.270	[3.637, 5.013]	<0.001
Developmental delay	0.229	0.206	1.257	[0.839, 1.883]	0.267
Learning disability	0.086	0.136	1.089	[0.834, 1.422]	0.529

Note. Reference category for outcome = no current depression. n = 63,924 unweighted.

**Table 5:** Comparison of adjusted odds ratios (95% CI) across no-NDD, Any-NDD, ADHD-only, ASD-only, and full-sample models.

Outcome/Predictor	No-NDD	Any-NDD	ADHD-Only	ASD-Only	Full Sample
Flourishing—Parent MH	0.61 [0.57, 0.65]	0.64 [0.56, 0.73]	0.69 [0.59, 0.80]	0.66 [0.51, 0.87]	0.62 [0.58, 0.65]
Flourishing—Bullying	0.65 [0.62, 0.69]	0.62 [0.58, 0.67]	0.61 [0.55, 0.67]	0.61 [0.50, 0.73]	0.65 [0.62, 0.68]
Anxiety—Parent MH	1.78 [1.64, 1.92]	1.45 [1.30, 1.61]	1.43 [1.26, 1.61]	1.52 [1.24, 1.86]	1.64 [1.54, 1.76]
Anxiety—Bullying	1.73 [1.63, 1.85]	1.52 [1.43, 1.62]	1.51 [1.40, 1.63]	1.51 [1.34, 1.70]	1.63 [1.56, 1.71]
Depression—Parent MH	2.04 [1.80, 2.32]	1.81 [1.56, 2.11]	1.84 [1.57, 2.16]	1.76 [1.38, 2.25]	1.95 [1.76, 2.17]
Depression—Bullying	1.96 [1.79, 2.15]	1.59 [1.48, 1.70]	1.58 [1.46, 1.71]	1.63 [1.44, 1.84]	1.75 [1.65, 1.85]

**Table 6:** Predicted probabilities (%) by bullying level and ADHD status.

Stratum	Never	Level 2	Level 3	Level 4	Daily	Δ (1→5), pp
Anxiety—without ADHD	5.7	11.6	23.1	27.5	34.6	+28.9
Anxiety—with ADHD	28.4	42.3	54.7	61.2	69.9	+41.5
Depression—without ADHD	1.9	3.9	8.8	11.9	21.7	+19.8
Depression—with ADHD	11.9	15.3	22.7	28.3	44.7	+32.8

## Data Availability

The 2023–2024 NSCH public-use data are publicly available from the U.S. Census Bureau and the Maternal and Child Health Bureau (https://mchb.hrsa.gov/data-research/national-survey-childrens-health accessed on 1 June 2026). CAHMI DRC-processed files are available at https://www.childhealthdata.org/ (accessed on 1 June 2026). SPSS analytic syntax and R code for the multiple imputation sensitivity analysis are available from the corresponding author upon reasonable request.
